# Evolutionary Dynamics and Emergence of Panzootic H5N1 Influenza Viruses

**DOI:** 10.1371/journal.ppat.1000161

**Published:** 2008-09-26

**Authors:** Dhanasekaran Vijaykrishna, Justin Bahl, Steven Riley, Lian Duan, Jin Xia Zhang, Honglin Chen, J. S. Malik Peiris, Gavin J. D. Smith, Yi Guan

**Affiliations:** 1 State Key Laboratory of Emerging Infectious Diseases, Department of Microbiology, Li Ka Shing Faculty of Medicine, The University of Hong Kong, Pokfulam, Special Administrative Region, China; 2 International Institute of Infection and Immunity, Shantou University Medical College, Shantou, Guangdong, China; 3 Department of Community Medicine and School of Public Health, Li Ka Shing Faculty of Medicine, The University of Hong Kong, Pokfulam, Hong Kong Special Administrative Region, China; Erasmus Medical Center, The Netherlands

## Abstract

The highly pathogenic avian influenza (HPAI) H5N1 virus lineage has undergone extensive genetic reassortment with viruses from different sources to produce numerous H5N1 genotypes, and also developed into multiple genetically distinct sublineages in China. From there, the virus has spread to over 60 countries. The ecological success of this virus in diverse species of both poultry and wild birds with frequent introduction to humans suggests that it is a likely source of the next human pandemic. Therefore, the evolutionary and ecological characteristics of its emergence from wild birds into poultry are of considerable interest. Here, we apply the latest analytical techniques to infer the early evolutionary dynamics of H5N1 virus in the population from which it emerged (wild birds and domestic poultry). By estimating the time of most recent common ancestors of each gene segment, we show that the H5N1 prototype virus was likely introduced from wild birds into poultry as a non-reassortant low pathogenic avian influenza H5N1 virus and was not generated by reassortment in poultry. In contrast, more recent H5N1 genotypes were generated locally in aquatic poultry after the prototype virus (A/goose/Guangdong/1/96) introduction occurred, i.e., they were not a result of additional emergence from wild birds. We show that the H5N1 virus was introduced into Indonesia and Vietnam 3–6 months prior to detection of the first outbreaks in those countries. Population dynamics analyses revealed a rapid increase in the genetic diversity of A/goose/Guangdong/1/96 lineage viruses from mid-1999 to early 2000. Our results suggest that the transmission of reassortant viruses through the mixed poultry population in farms and markets in China has selected HPAI H5N1 viruses that are well adapted to multiple hosts and reduced the interspecies transmission barrier of those viruses.

## Author Summary

H5N1 influenza virus has been responsible for poultry outbreaks over the last 12 years—the longest recorded example of highly pathogenic avian influenza (HPAI) circulation in poultry. The ecological success of this virus in diverse species of both poultry and wild birds with sporadic introduction to humans suggests that it is a likely source of the next human pandemic. Genome sequences of H5N1 viruses reveal extensive genetic reassortment (mixing) with other influenza subtypes to produce many H5N1 genotypes that have developed into multiple genetically distinct clades, some of which have spread to affect over 60 countries. Here, we analyze all available sequence data of avian influenza viruses from Eurasia and show that the original HPAI H5N1 virus (referred to as A/goose/Guangdong/1/96) was likely introduced directly into poultry as an intact virus particle from wild aquatic birds. In contrast, H5N1 genotypes were generated in aquatic poultry populations after the introduction of A/goose/Guangdong/1/96 virus. Our results suggest that the transmission of reassortant viruses through the diverse poultry populations in farms and markets in China has selected H5N1 viruses that are well-adapted to multiple hosts and reduced the interspecies transmission barrier of those viruses.

## Introduction

Outbreaks of highly pathogenic avian influenza (HPAI) H5N1 virus were first recorded in Guangdong, China in 1996 [1]. Since its emergence, the A/goose/Guangdong/1/96 (Gs/GD) virus lineage has become the longest recorded HPAI virus to remain endemic in poultry [2]. The ecological success of this virus in diverse avian and mammalian species [3] with frequent introduction to humans suggests this virus is the most likely candidate of the next human pandemic [4]. Therefore, the evolutionary and ecological characteristics of its emergence from wild birds into poultry are of considerable interest.

The virus gradually became endemic in poultry in different regions of China, developing into genetically and antigenically distinct sublineages [5],[6]. The geographic spread of these sublineages outside China is also unprecedented, with two sublineages spreading to Southeast Asia in late 2003, and another westwards to Central Asia, Europe, Africa, the Middle East and the Indian subcontinent in mid-2005 [1], [5]–[9]. During mid-2005, one sublineage (Fujian-like or clade 2.3.4) became dominant in China and subsequently spread to Laos, Thailand and Vietnam [2],[10].

Influenza surveillance in southern China has revealed that the Gs/GD virus lineage underwent extensive genetic reassortment to generate many different reassortant viruses (or genotypes) between 1997 and 2006 [5],[6]. The non-reassortant Gs/GD-like viruses were prevalent only from 1996 to 2000 [11]. Afterwards, all H5N1 viruses detected were reassortant genotypes. Amongst all recognized reassortants, only genotypes B, X0, W, Z, G and V, were persistent for more than two years or predominant at different time points, while many genotypes were only detected occasionally [5],[6].

While the genetic and antigenic evolution and geographic spread of the HPAI H5N1 panzootic viruses are well documented after the initiation of systematic surveillance in 2000 [5], little is known about the source and early evolutionary dynamics of H5N1 virus. In particular, it is still unknown whether the Gs/GD virus itself was a reassortant virus or introduced wholly from migratory waterfowl. Even though the internal gene sources for most genotypes have been identified from aquatic birds [12], the order of emergence and reassortment events that generated these genotypes remains to be answered.

Recently, Dugan et al. [13] described an evolutionary dynamics of avian influenza virus (AIV) in wild bird populations. They found that the viruses exist as a large pool of functionally equivalent genes, under strong purifying selection, and reassort without overall loss of virus fitness resulting in many transient genotypes. Other studies have shown that all eight gene segments of the HPAI H5N1 viruses exhibit rapid nucleotide and amino acid substitution rates in comparison to other subtypes and that reassortment with the wild bird AIV gene pool has increased H5N1 genotype diversity, producing both persistent and transient genotypes [5],[12],[14]. However, the mechanism behind the population behavior of H5N1 viruses has still not been investigated. Here we aim to address these gaps in our knowledge of the evolutionary dynamics of HPAI H5N1 viruses through the estimation of divergence times of gene segments of major reassortants and population dynamics analyses of H5N1 viruses in poultry.

## Results

### Study design

In the present study, all H5N1 influenza viruses with a hemagglutinin gene derived from A/goose/Guangdong/1/96 were designated as belonging to the “Gs/GD lineage”. The H5N1 reassortants from the Gs/GD lineage were referred to by their genotype designation, such as B, X0, Z, V, W and G, as described previously [5],[6]. Non-reassortant viruses with all eight gene segments closely related to A/goose/Guangdong/1/96 were referred to as “Gs/GD-like”.

Our results are presented in four parts: 1) estimation of the time of emergence of the Gs/GD-like virus; 2) estimation of the dates of emergence of genotypes; 3) estimation of the dates of emergence of H5N1 clades; and 4) description of the evolutionary dynamics of HPAI H5N1 viruses in poultry in China. Phylogenetic analysis was conducted using relaxed clock and Bayesian skyline coalescent models [15],[16] as implemented in the BEAST software package version 1.4.7 [17].

First, dates of divergence of the H5-hemagglutinin (HA) and N1-neuraminidase (NA) genes of representative Eurasian viruses were estimated. Due to the large number of Gs/GD-like H5N1 virus sequences available, a representative subset of H5N1 sequences were aligned with all publicly available avian H5-HA and N1-NA sequences. The final datasets consisted of 116 H5-HA and 89 N1-NA genes of 1,014 and 1,356 nucleotides in length, respectively, and are referred to as “Eurasian” datasets. Because the exact isolation dates of many viruses were not available, the mid-years of virus isolation were used as calibration points.

For dating the emergence of the internal genes of the GsGD-like virus and of the H5N1 genotypes, we used internal gene datasets (referred to as “Asian” datasets) that included representative viruses of all identified H5N1 genotypes, plus viruses of other subtypes from poultry and wild aquatic birds. The mid-years of virus isolation were used as calibration points to estimate the time of incorporation of novel gene segments into Gs/GD-like genotypes. Because the non-structural (NS) genes of Gs/GD-like viruses (allele A) and most of its reassortants (allele B) are highly divergent [1], the time of most recent common ancestor (TMRCA) for each allele were calculated separately.

To estimate the dates of emergence of different Gs/GD-like H5N1 clades, H5-HA datasets were recruited that contained representative viruses of known H5N1 variants. The final dataset consisted of the complete HA gene (1,733 nucleotides in length) of 192 viruses, and is also referred to as the “Asian” H5-HA dataset. The exact dates of sampling were known for most of these samples and were used as calibration time points. For those sequences for which exact virus isolation dates were not available, the mid-month date (either the 14th or 15th) was used as the calibration point. However, in some instances, only the date of initial detection of an H5N1 outbreak was available and this was used for calibration [18].

Finally, to examine changes in genetic diversity during the evolution of the Gs/GD lineage in China, we constructed Bayesian skyline plots of the virus population described by a modified Asian H5-HA dataset [19]. This dataset consisted of HA genes of viruses isolated from chicken (n = 54), duck (n = 52), goose (n = 15), pheasant (1) and Guinea fowl (1) in China.

### Rates of nucleotide substitution

Our analyses showed that the mean substitution rates for the H5 and N1 datasets were 4.77×10^−3^ and 5.19×10^−3^ substitutions per site, per year (subst/site/year), respectively, which were significantly higher than the rates of the internal gene segments (1.84–2.62×10^−3^ subst/site/year) (Table 1). These rates are similar to those previously described for avian, human, equine and swine influenza viruses [14],[20],[21].

**Table 1:** Best-fit relaxed clock model and mean nucleotide substitution rates

Dataset	Gene	Uncorrelated relaxed clock model	Mean substitution rate (×10^−3^)	Substitution rate HPD (×10^−3^)
Eurasian	H5	exponential	4.77	3.88–5.74
	N1	exponential	5.19	4.17–6.17
Asian	PB2	lognormal	2.41	1.92–2.90
	PB1	lognormal	2.59	2.13–3.04
	PA	exponential	2.62	1.9–3.2
	NP	exponential	2.47	1.9–3.03
	M	exponential	1.84	1.3–2.30
	NS	exponential	2.51	1.77–3.44
	H5	exponential	4.23	3.58–4.93
	N1	exponential	4.27	3.27–5.25

### Emergence of the Gs/GD virus

**Figure 1:**
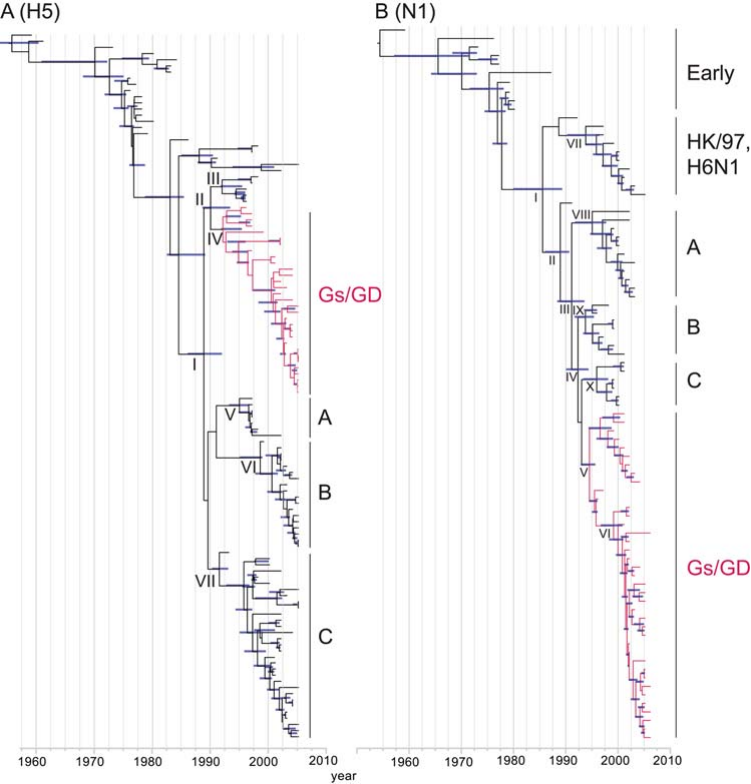
Dated phylogeny of the surface genes of H5N1 viruses isolated in Eurasia. The HA gene (A) and N1 gene (B) trees scaled to time (horizontal axis) generated using the SRD06 codon model and uncorrelated relaxed clock model. Nodes correspond to mean TMRCAs and blue horizontal bars at nodes represent the 95% HPDs of TMRCAs. Red branches indicate Gs/GD lineage H5N1 viruses. Identical phylogenetic trees with virus names are shown in Figure S1. TMRCAs and HPDs for each of the nodes marked with Roman numerals are given in Table 2.

Analysis of the Eurasian H5-HA dataset revealed four distinct H5 lineages currently circulating in Eurasia: the Gs/GD lineage plus two lineages exclusively of European virus isolates (groups A and B) and one containing isolates from throughout Eurasia (group C) (Figure 1A, S1A). The time of divergence of these four lineages (node I) was Jul 1989 (95% highest posterior density Jul 1986–Jun 1992) (Figure 1A; Table 2). The TMRCA for the Gs/GD lineage (node IV) was estimated to be Jan 1994 (95% highest posterior density (HPD) Apr 1992–Nov 1995) (Figure 1A; Table 2). The TMRCAs of the remaining groups are presented in Table 2.

**Table 2:** Estimated TMRCAs for the Eurasian H5-HA and N1-NA datasets

Dataset	Node^1^	Description	Mean TMRCA (95% HPD)^2^
H5 HA dataset	I	Current Eurasia	1989.51 (1986.52–1992.42)
	II	Gs/GD+out-group	1991.69 (1989.06–1993.83)
	III	Hokkaido/Singapore	1994.21 (1992.19–1995.91)
	IV	Gs/GD H5N1	1994.04 (1992.27–1995.84)
	V	Europe A	1995.64 (1993.64–1997.06)
	VI	Europe B	1997.88 (1995.47–1999.39)
	VII	Eurasia C	1992.48 (1992.71–1993.49)
N1 NA dataset	I	Current Eurasia	1985.83 (1980.32–1989.67)
	II	Eurasia	1989.21 (1986.30–1991.01)
	III	Eurasia	1991.40 (1988.73–1993.94)
	IV	Eurasia	1992.59 (1990.45–1994.71)
	V	GsGD	1994.81 (1993.23–1996.02)
	VI	GsGD with 20 AA deletion	1999.42 (1997.05–2001.23)
	VII	HK97	1994.08 (1990.61–1996.79)
	VIII	Eurasia A	1995.33 (1992.10–1998.08)
	IX	Eurasia B	1994.03 (1992.04–1995.75)
	X	Eurasia C	1996.19 (1993.68–1998.49)

1 Nodes indicate TMRCAs of major H5 and N1 lineages, as shown in Figures 1A, B.

2 The dates are presented as year followed by proportion of days.

**Figure 2:**
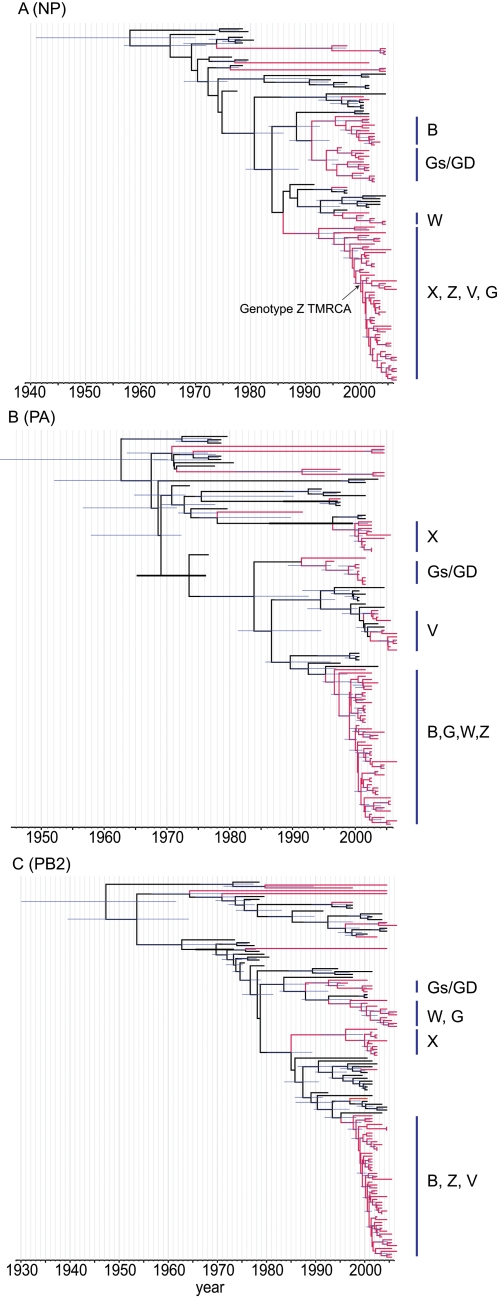
Dated phylogeny of the internal genes of viruses isolated in Asia. The NP (A), PA (B) and PB2 (C) gene trees scaled to time (horizontal axis) generated using the SRD06 codon model and uncorrelated relaxed clock model. Nodes correspond to mean TMRCAs and blue horizontal bars at nodes represent the 95% HPDs of TMRCAs. Red branches indicate Gs/GD lineage H5N1 viruses. Identical phylogenetic trees with virus names are shown in Figure S2. TMRCAs and HPDs for each of the major H5N1 genotype internal genes are given in Table 3.

Similarly, multiple N1-NA lineages were also recognized in Eurasia (Figure 1B, S1B). The TMRCA for the Gs/GD lineage NA (node V) was estimated to be Oct 1994 (HPD Mar 1993–Jan 1996) (Figure 1B; Table 2). It was noted that the N1 gene of the HK/97-like H5N1 virus clustered along with the NA genes of H6N1 viruses from poultry (node VII), with an estimated TMRCA of Jan 1994 (HPD Aug 1990–Oct 1996) (Figure 1B; Table 2). The TMRCA for each of the internal gene segments of the Gs/GD-like viruses were estimated using the Asian datasets and ranged from Jul 1993 for the polymerase acidic (PA) gene to May 1995 for the NS gene (Figure 2 and Table 3).

**Table 3:** TMRCAs of internal gene segments of H5N1 viruses

	GsGD (Gs/GD/1/1996)	X (Dk/ST/4912/2001)	B (Ck/HK/YU562/01)	W (Dk/Zhejiang/52/00)	Z (Ck/HK/YU22/02)	V (B.bird/Hunan/1/04)	G (Dk/Guangxi/13/04)
PB2	1994.34 (1992.01–1995.95)	1996.04 (1991.11–1999.75)	1997.58 (1995.63–1999.39)	1996.96 (1993.12–2000.19)	B*	B	W
PB1	1994.77 (1993.62–1995.71)	2000.41 (1999.37–2001.26)	1995.84 (1993.1–1998.27)	B	B	B	B
PA	1993.52 (1989.21–1996.05)	1999.16 (1996.56–2000.88)	1997.29 (1994.73–1999.29)	B	B	2002.18 (2001.03–2003.06)	B
NP	1993.67 (1990.46–1995.94)	1992.29 (1988.19–1996.43)	1995.32 (1991.69–1998.41)	1995.08 (1992.21–1997.06)	1999.94 (1998.67–2001.06)	X0	X0
M	1994.23 (1990.7–1996.36)	1999.49 (1997.42–2001.08)	1997.83 (1995.58–1999.7)	B	B	B	B
NS	1995.35 (1992.33–1997.29)	1999.31 (1997.25–2001.14)	1996.88 (1993.7–1999.46)	B	B	B	B

*: Refers to the gene being derived from an earlier H5N1 genotype.

Bayes factor (BF) tests showed no significant difference between TMRCAs of all eight gene segments of the Gs/GD viruses (Table S1). These results indicate that the common ancestor of the Gs/GD-like virus was generated, probably in wild waterfowl, approximately 2 years before it was first detected in poultry in 1996.

### Generation of H5N1 reassortant viruses

**Figure 3:**
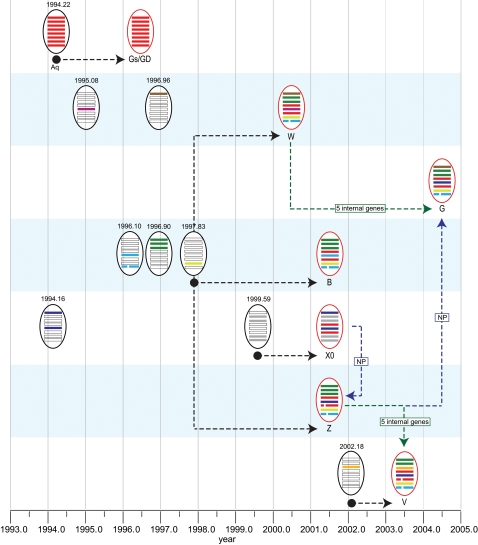
Diagram representing the emergence of major H5N1 reassortant viruses. Virus particles outlined in black represent donor viruses (with mean TMRCAs above the particle) and those outlined in red represent characterized H5N1 genotypes placed at the year of first detection. Gene segments are ordered PB2, PB1, PA, HA, NP, NA, M and NS from top to bottom within the virus particle diagram. Arrows represent possible reassortment pathways of genotype development. The start of the black arrows (filled circles) indicate the earliest possible time of corresponding genotype generation. Colored arrows represent reassortment between existing H5N1 genotypes.

The TMRCAs of the internal gene segments for each major H5N1 genotype (B, X0, W, Z, G and V) were estimated using the Asian datasets from which we inferred the dates of emergence of these genotypes (Figures 2 and 3; Table 3). The mean TMRCAs for the internal genes of genotype B virus ranged from Apr 1995 to Nov 1997 (Table 3). Bayes factor tests showed that the internal genes of genotype B were incorporated from three different sources (Table S1). This suggests that the three internal gene segments of genotype B virus (polymerase basic 2 (PB2), polymerase basic 1 (PB1), polymerase acidic (PA)) were derived from the same virus (mean TMRCA range Nov 1995–Jul 1997), while the NP and NS genes (mean TMRCA range Apr 1995–Nov 1996) was from a different source (Table 3), while the M gene (mean TMRCA Nov 1997) was from an independent sources. Therefore, genotype B virus was generated from viruses of four independent sources, i.e. it contains surface genes from Gs/GD and internal genes from three other sources. The mean TMRCA of the last incorporated sources (M gene) was Nov 1997. This date represents the earliest possible time when all genotype B internal gene segments co-circulated in the poultry gene pool in China. Therefore, genotype B virus was generated after mid-1997 (Figure 3).

Both genotypes B and W appear to have emerged during the same period, i.e. after mid-1997 (Figure 3). Genotype W shares four internal gene segments (PB1, PA, M and NS) with genotype B, while its PB2 and NP segments are from two different aquatic sources (Figures 2 and S2). The TMRCAs for the PB2 and NP genes were estimated at Dec 1996 (HPD Feb 1993–Mar 2000) and Jan 1995 (HPD Mar 1992–Jan 1997), respectively, and these dates were significantly different (Tables 3 and S1). These results suggest that genotype W has been generated through a reassortment of viruses from six different sources.

However, the estimated number of gene sources for genotypes B and W should be treated with caution. As the mid-year of isolation (e.g. 2000.5) was used to calibrate the internal gene datasets, the extension of confidence intervals to account for this error indicates that the three internal gene sources for genotype B may not be significantly different. The availability of the date of isolation for viruses would be needed to estimate the age of gene sources for genotypes B and W with greater confidence.

Genotype X0 does not share any internal genes with genotypes B and W, and has incorporated internal genes from two different sources, i.e. the PB1, PA, M and NS genes were from one source, and while PB2 and NP genes are from another (Figures 2 and S2). For genotype X0 virus, the mean TMRCA range of the internal genes was Apr 1992 to May 2000 (Table 3). The TMRCAs of the PB1, PA, M and NS genes were not significantly different (Table S1). However, the TMRCAs of the NP and PB2 genes were significantly earlier than the other genes, but without significant difference between them, suggesting a common source. The averaged TMRCA means of the five most recent internal genes (Jul 1999) indicates that genotype X0 was generated after mid 1999 (Table 3 and Figure 3).

The predominant genotype Z virus was derived from genotype B, with which it shares five internal genes (PB2, PB1, PA, M and NS). The NP gene has a common source with genotype X0 viruses. The TMRCA of the NP gene (Figure 2A) was estimated at Dec 1999 (HPD Sep 1998–Jan 2001), indicating that genotype Z emerged late 1999/early 2000 (Figure 3 and Table 3). Genotype V virus shares all internal genes with genotype Z except for the PA gene. The PA gene of genotype V viruses are most closely related to viruses isolated from the aquatic gene pool and therefore is most likely derived from an aquatic source (Figure 2B). The TMRCA for the PA gene was estimated as Mar 2002 (HPD Jan 2001–Jan 2003), suggesting genotype V was generated after early-2002 (Table 3). Genotype G viruses have five internal genes (PB2, PB1, PA, M and NS) in common with genotype W, while the NP gene groups with those from genotype Z and V viruses (Figures 2 and 3). Therefore, it was not possible to estimate the date of emergence of genotype G virus.

### Geographical expansion of H5N1

**Figure 4:**
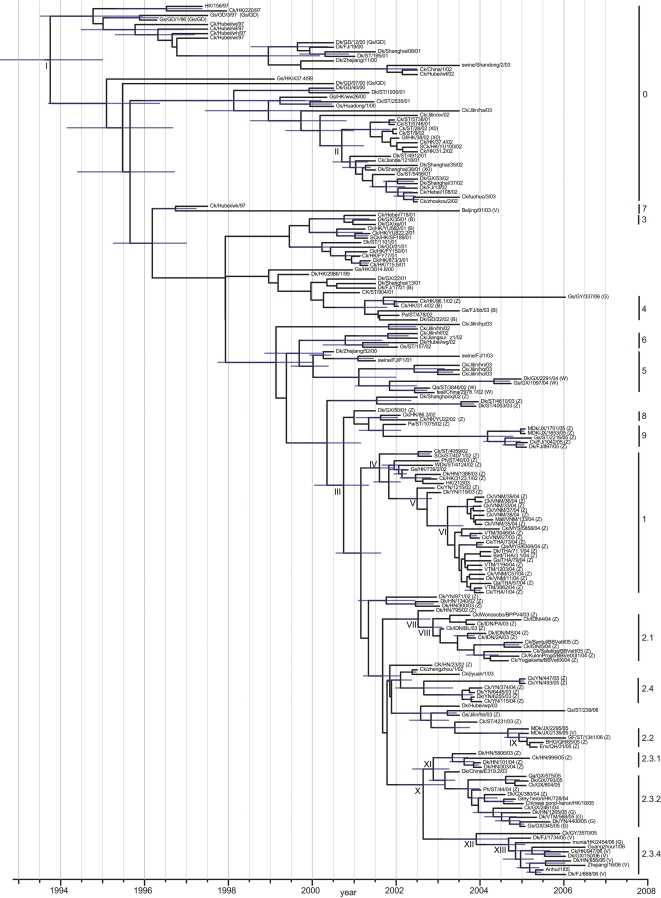
Dated phylogeny of the HA gene of H5N1 viruses isolated in Asia. The tree is scaled to time (horizontal axis) and was generated using the SRD06 codon model and uncorrelated relaxed clock model. Nodes correspond to mean TMRCAs and blue horizontal bars at nodes represent the 95% HPDs of TMRCAs. TMRCAs and HPDs for each of the major H5N1 lineage are given in Table 4.

The introduction to Indonesia occurred approximately 3–4 months before the introduction to Vietnam and Thailand (Figure 4 and Table 4). The TMRCA for viruses isolated from Vietnam, Thailand and Malaysia (clade 1, node VI) was estimated at Mar 2003 (HPD Oct 2002–Aug 2003), while the TMRCA for the Indonesia viruses (clade 2.1, node VIII) was estimated at Nov 2002 (HPD Jul 2002–Feb 2003) (Figure 4). A BF test indicated these dates were significantly different. Both of these TMRCAs are approximately 3–6 months before the first observed H5N1 outbreaks in the field in these countries.

**Table 4:** Estimated TMRCAs for H5N1 HA clades

Nodes	Description	TMRCA (95% HPD)
I	GsGD	1993.75 (1992.50–1994.96)
II	X-series	2000.70 (2000.11–2001.18)
III	Clades 1, 2, 8, 9	2000.74 (2000.06–2001.36)
IV	Clade 1	2001.16 (2000.57–2001.66)
V	Vietnam, Thailand, Malaysia (VTM)+precursor	2000.74 (2000.06–2001.36)
VI	VTM	2003.22 (2002.80–2003.62)
VII	Indonesia+precursor	2002.52 (2002.09–2002.84)
VIII	Clade 2.1 (Indonesia)	2002.87 (2002.58–2003.12)
IX	Clade 2.2 (Qinghai lineage)	2004.93 (2004.59–2005.14)
X	Clade 2.3	2002.64 (2002.06–2003.16)
XI	Clades 2.3.1, 2.3.2	2002.88 (2002.39–2003.29)
XII	Clades 2.3.3, 2.3.4	2003.91 (2003.11–2004.70)
XIII	Clade 2.3.4 (Fujian-like)	2004.68 (2004.17–2005.09)

The TMRCA of the Qinghai-like HA variant (clade 2.2, node IX) was estimated as Dec 2004 (HPD Aug 2004–Feb 2005), while the Fujian-like HA TMRCA (clade 2.3.4, node XIII) was estimated as Sep 2004 (Mar 2004–Feb 2005) (Figure 4). These TMRCAs were not significantly different (Table S1), indicating that these two variants arose during the same period.

### Estimation of genetic diversity and selection pressure

**Figure 5:**
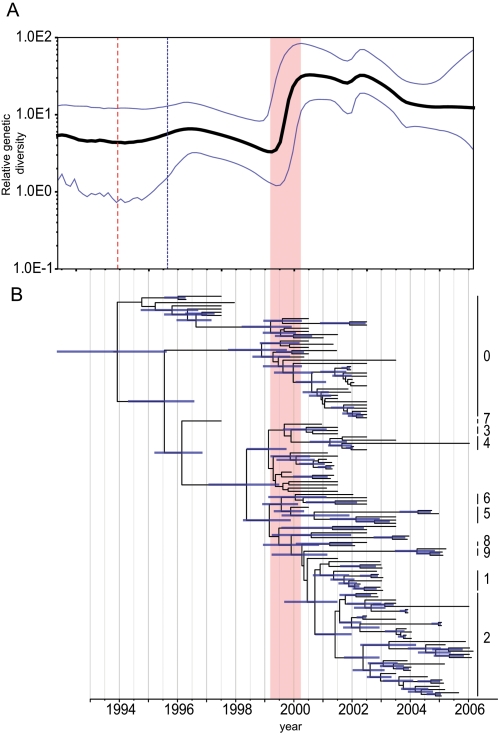
Population dynamics of genetic diversity of H5N1 viruses isolated from poultry in China. Bayesian skyline plot of the HA gene (A) showing changes in genetic diversity of H5N1 viruses. A measure of genetic diversity is given on the y-axis with the 95% HPD shown in blue. The red dashed line indicates the mean TMRCA of the Gs/GD lineage; the blue dashed line represents the time of the first detected H5N1 outbreak China. HA gene tree (B) scaled to time (horizontal axis) generated using the SRD06 codon model and uncorrelated relaxed clock model. Nodes correspond to mean TMRCAs and blue horizontal bars at nodes represent the 95% HPDs of TMRCAs. Numbers to the right of the HA tree indicate H5N1 clades based on the World Health Organization nomenclature system [36]. An identical phylogenetic tree with virus names is shown in Figure S3. The red vertical bar in both panels indicates the period of divergence of major H5N1 lineages in poultry.

Coalescent reconstruction using a HA-H5 dataset of poultry isolated in China revealed a rapid increase in the relative genetic diversity of Gs/GD lineage viruses from mid-1999 to 2000 (Figure 5A). It was during this period, preceding the current epizootic, that each of the major HA lineages were generated and subsequently became widespread in poultry throughout China (Figure 5B).

Analysis of selection pressures show that the HA of all the major H5N1 sublineages (or clades) were subject to strong purifying selection (mean ratio of rates of nonsynonymous to synonymous substitutions per site, *d*_N_/*d*_S_ = 0.272). However, four amino acid sites (positions 138, 140, 141 and 156) were under positive selection (*P*<0.05), consistent with previous results for AIV [14],[22],[23].

## Discussion

We used extensive sequence data and relaxed clock models to date each of the eight gene segments of the Gs/GD-like H5N1 virus. Previously, phylogenetic analyses have shown that the gene segments of Gs/GD-like viruses were most closely related to those viruses from migratory birds [12]. However, the time of introduction into poultry was not established. We have shown that the prototype virus was likely introduced into poultry as a non-reassortant low pathogenic avian influenza H5N1 virus, rather than being generated by reassortment within poultry. We have also shown that the reassortment events that generated these H5N1 genotypes occurred locally in domestic duck after the Gs/GD-like virus introduction (Figure 3).

Our data help provide insights into the sequence of events that led to each of the three H5N1 transmission waves. Previously, the earliest records of H5N1 wave 1 outbreaks in Vietnam and Indonesia were August and October 2003, respectively [22]. However, estimation of the TMRCAs of these two lineages indicates that H5N1 virus introduction to Indonesia occurred approximately 3–4 months before the introduction to Vietnam. Furthermore, both of these estimated dates of introduction are approximately 3–6 months before the first observed H5N1 outbreaks in these countries. This may represent the time for disease development and under reporting once outbreaks occurred. In contrast, the TMRCAs of the wave 2 and 3 H5N1 viruses indicated that these HA variants arose during the same period in late 2004.

Analysis of virus population dynamics revealed a rapid increase in the genetic diversity of Gs/GD lineage in poultry in China from mid-1999 to early 2000. This corresponds with the period when each of the major Gs/GD-like H5N1 variants or sublineages diverged and subsequently became widespread in poultry throughout China [6]. It is likely that combined strong ecological and evolutionary factors led to this rapid increase in diversity, namely, the spread of the virus through large, immunologically naive poultry populations consisting of diverse species coupled with relatively high rates of nucleotide substitution and selection pressure in the HA. Interestingly, the first detection of current H5N1 reassortants occurred in early 2000, some time after the 1997 Hong Kong ‘Bird Flu’ outbreak in poultry occurred [11]. This leads us to hypothesize that these reassortant viruses, particularly genotypes B, X0, W and Z, were also generated during this period. This hypothesis is supported by our field data in which different H5N1 genotype viruses have been isolated from the same market on the same sampling occasion [24],[25].

We also explored a possible mechanism to explain the population behavior of this virus, particularly the generation and maintenance of multiple H5N1 reassortants. The frequent reassortment of the polymerase complex (PB2, PB1, PA and NP) and matrix genes observed in H5N1 viruses indicates that the fitness landscape is similar to that observed for AIV in their natural gene pools, wherein little or no change in fitness is associated with frequent reassortment of functionally equivalent gene segments [5],[6],[13]. The presence of most of these internal genes in domestic duck before their detection in H5N1 genotypes suggests that reassortment occurred in these hosts [11].

We propose a specific mechanism to explain observed patterns of genetic drift and reassortment in H5N1. First, AIV (of different subtypes) from the natural gene pool in wild birds are introduced into domestic duck. In domestic duck, these viruses undergo regular reassortment with endemic H5N1 viruses. Subsequently, transmission of these reassortant viruses within large highly connected populations of duck and other poultry species results in frequent interspecies transmission and genetic drift. Therefore, it is likely that this process selects for relatively fit viruses with a broad host range which are subsequently exported to other geographical regions. It is interesting to note that further reassortment has not been observed once those H5N1 viruses were transmitted out of China. We suggest that host population structures elsewhere may not result in the same intense multi-species transmission we observe in southern China.

## Methods

### Virus and viral sequence data

We sequenced the full genome of 31 low pathogenic avian influenza H5 viruses plus 135 HPAI H5N1 viruses isolated from our surveillance program in southern China or otherwise available in our repository (GenBank accession numbers CY028924–CY029507, CY030878–CY031006, EF597247–EF597498). A further 114 partial gene sequences from 29 H5N1 and other subtype viruses isolated from 2000–2005 were also resequenced to provide full-length genomes. These sequences were analyzed together with all publicly available sequence data, including genes from 93 H6 and 279 H9 viruses that were also recently sequenced in our laboratories. Viral genome sequencing was conducted as previously described [2],[24].

### Phylogenetic reconstruction and Bayesian skyline plots

To estimate the times of divergence, a total of nine full-length datasets were analyzed: two Eurasian datasets for the H5-HA and N1-NA, and seven Asian datasets for each of the influenza gene segments, except the NA gene. To examine changes in genetic diversity during the evolution of the Gs/GD lineage, we constructed Bayesian skyline plots using a modified Asian HA dataset. This modified dataset consisted of only HA genes of viruses isolated from chicken (n = 54), duck (n = 52), goose (n = 15), pheasant (1) and Guinea fowl (1) in China.

To estimate the rates of nucleotide substitution and TMRCAs, we used a Bayesian Markov chain Monte Carlo (MCMC) method as implemented in the program BEAST v1.4.7 [17],[26]. Each gene was analyzed with the codon based SRD06 nucleotide substitution model [27]. For each analysis the Bayesian skyline coalescent model was used [16]. Three clock models were compared statistically for each dataset using a Bayes factor test in Tracer v1.4 [28],[29]: the strict clock that assumes a single evolutionary rate along all branches, and the uncorrelated lognormal relaxed (UCLD) clock and uncorrelated exponential relaxed (UCED) clock that allow evolutionary rates to vary along branches within lognormal and exponential distributions, respectively [15]. A Bayes factor test of clock models showed that the UCED clock was most appropriate for datasets other than PB2 and PB1, for which the UCLD clock most appropriately described the data. For each dataset, three to five independent Bayesian MCMC runs were conducted for 10–20 million generations sampled to produce 10,000 trees. Convergence of the runs was confirmed using Tracer v1.4 [29] and effective sample size values of >500 indicated a sufficient level of sampling. The results of the multiple runs were then combined using LogCombiner v1.4.7 [17]. Mean evolutionary rates and divergence times were calculated using TreeAnnotator v1.4.7 and TreeStat v1.1 after the removal of an appropriate burnin, 10–20% in most cases, and phylogenetic trees were visualized with FigTree v1.1.2 [17],[30],[31].

Finally, to evaluate if the TMRCAs of each of the gene segments of a given genotype were significantly different or not, the TMRCA of each gene segment was compared to the remaining genes of the genotype using a Bayes factor test [28]. This test was calculated in as follows; given a genotype, the probability of any gene (e.g. PB2) being older than any other segment (e.g. PB1) divided by the probability of PB1 being older than PB2 given the data (tree estimates of TMRCA) multiplied by the inverse estimation for the priors (PB1 being older than PB2 divided by PB2 being older that PB1 of the priors) was calculated for each Bayesian MCMC run [19].

### Detection of selection pressure

To determine selection pressures on the HA of Gs/GD-like H5N1 viruses in poultry the modified Asian H5-HA dataset was analyzed using the single-likelihood ancestor counting (SLAC) [32] and genetic algorithm (GA) methods [33] available in DataMonkey [34] and HYPHY [35]. The SLAC method calculates global and site-specific nonsynonymous (*d*_N_) and synonymous (*d*_S_) nucleotide substitution rate ratios (ω = *d*_N_ */d*_S_) based on the BEAST generated phylogenetic tree and the best-fit nucleotide substitution model. The GA method assigns four ω classes to each lineage in search of the model of lineage-specific evolution that best fits the data [33]. The probability (≥95%) of ω being >1 along a specific lineage was calculated from the averaged model probability of all models rather than by inference from the single best-fitting model [33]. This approach does not require any *a priori* hypothesis of lineage-specific evolution.

## Supporting Information

Figure S1 The HA gene (A) and N1 gene (B) trees scaled to time (horizontal axis) generated using the SRD06 codon model and uncorrelated relaxed clock model. Nodes correspond to mean TMRCAs and blue horizontal bars at nodes represent the 95% HPDs of TMRCAs. Red branches indicate Gs/GD lineage H5N1 viruses.
(1.33 MB PDF)

Figure S2 The PB2 (A), PB1 (B), PA (C), NP (D), M (E) and NS (F) gene trees scaled to time (horizontal axis) generated using the SRD06 codon model and uncorrelated relaxed clock model. Nodes correspond to mean TMRCAs and blue horizontal bars at nodes represent the 95% HPDs of TMRCAs. Red branches indicate Gs/GD lineage H5N1 viruses.
(1.65 MB PDF)

Figure S3 HA gene tree of H5N1 viruses isolated from poultry in China, scaled to time (horizontal axis) generated using the SRD06 codon model and uncorrelated relaxed clock model. Nodes correspond to mean TMRCAs and blue horizontal bars at nodes represent the 95% HPDs of TMRCAs.
(0.43 MB PDF)

Table S1 (0.07 MB PDF)
