# Supplementary figures and images for: Evolutionary Dynamics and Emergence of Panzootic H5N1 Influenza Viruses
Source: PLoS Pathog. 2008 Sep 26;4(9):e1000161. doi: 10.1371/journal.ppat.1000161 (PMC2533123; doi:10.1371/journal.ppat.1000161)

Figure S1 A (Eurasian H5)

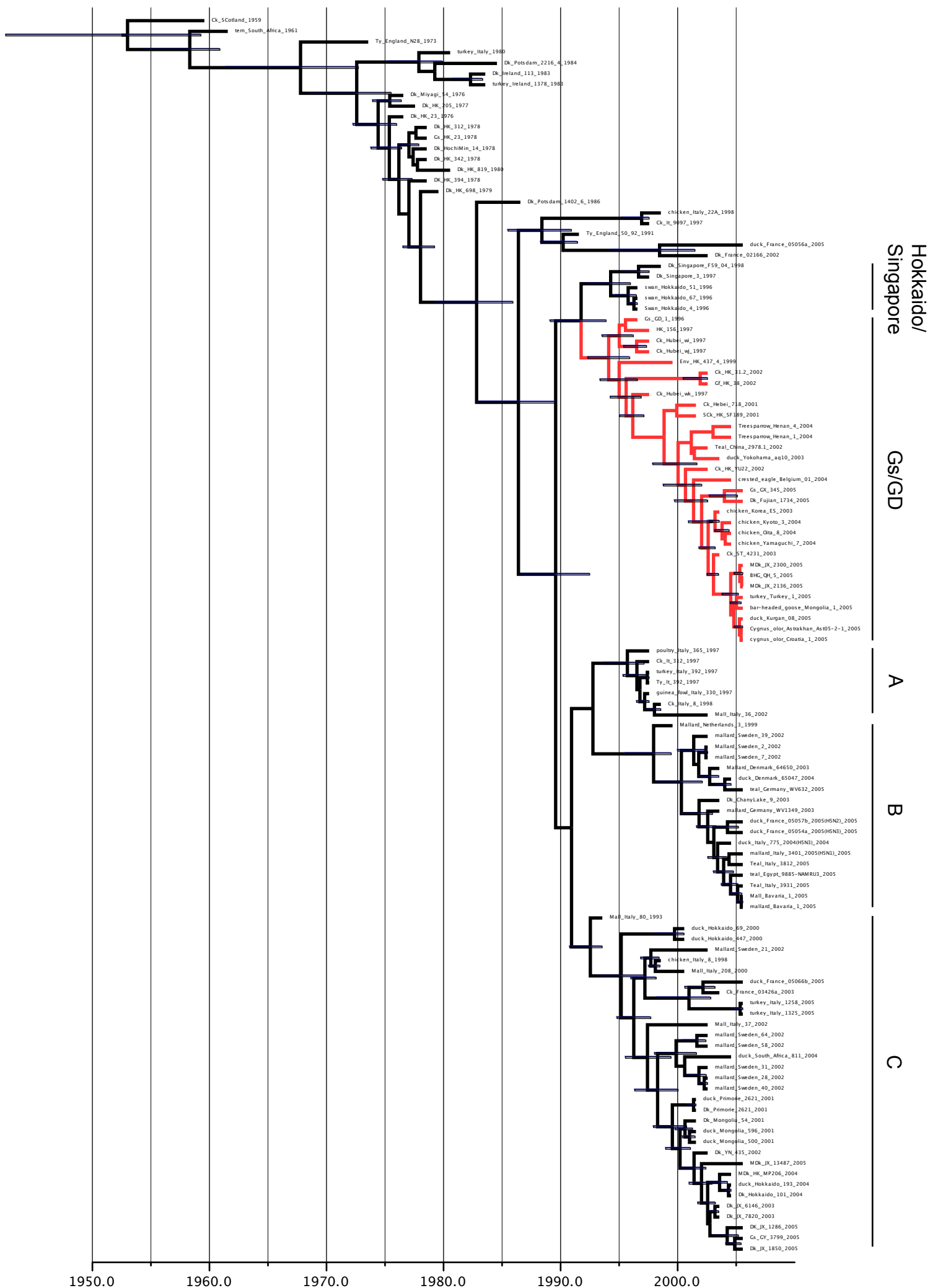

Figure S1 B (Eurasian N1)

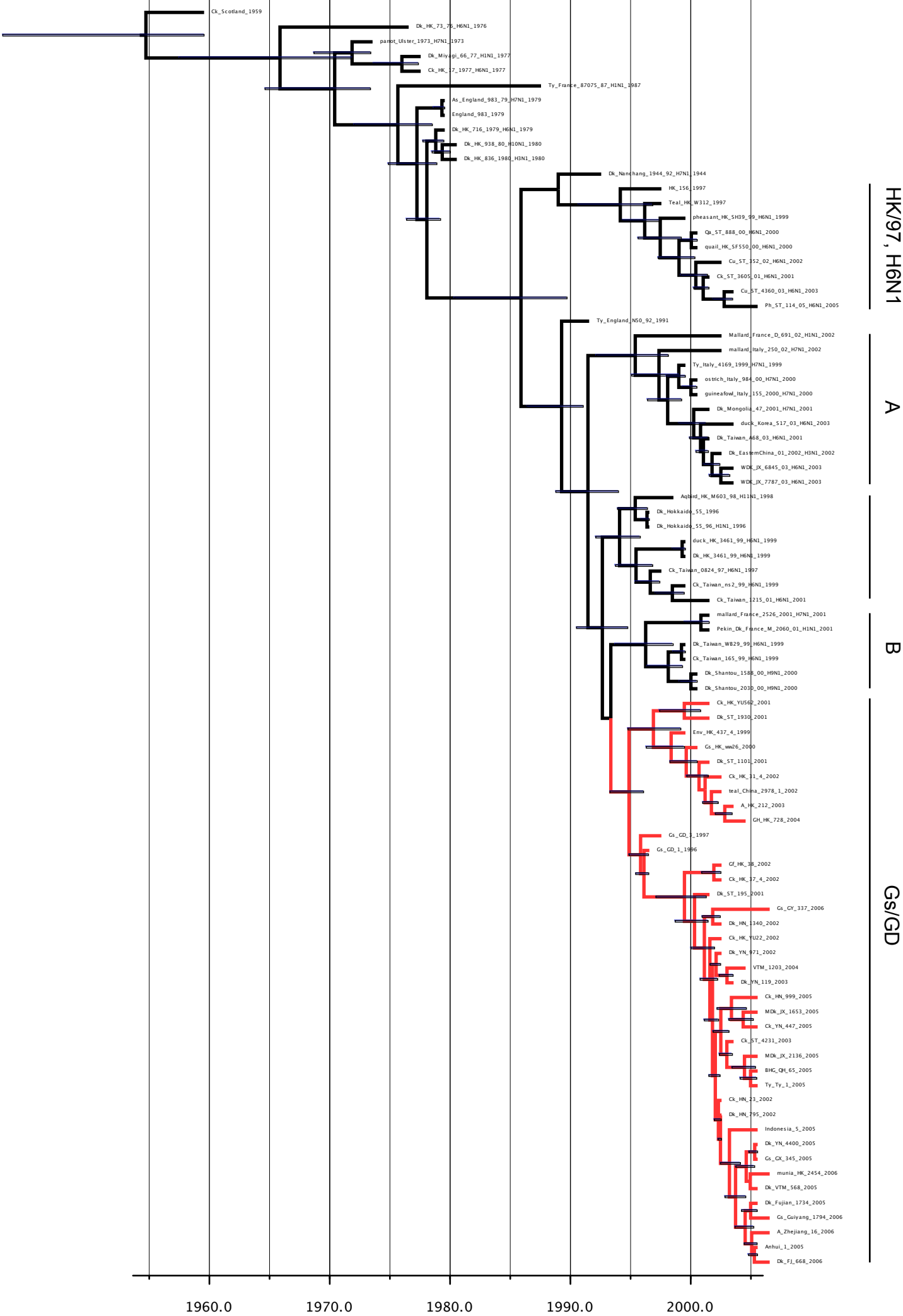

Supplement: Figure S1 — The HA gene (A) and N1 gene (B) trees scaled to time (horizontal axis) generated using the SRD06 codon model and uncorrelated relaxed clock model. Nodes correspond to mean TMRCAs and blue horizontal bars at nodes represent the 95% HPDs of TMRCAs. Red branches indicate Gs/GD lineage H5N1 viruses. (1.33 MB PDF) [file ppat.1000161.s001.pdf]

Figure S2 A (PB2)

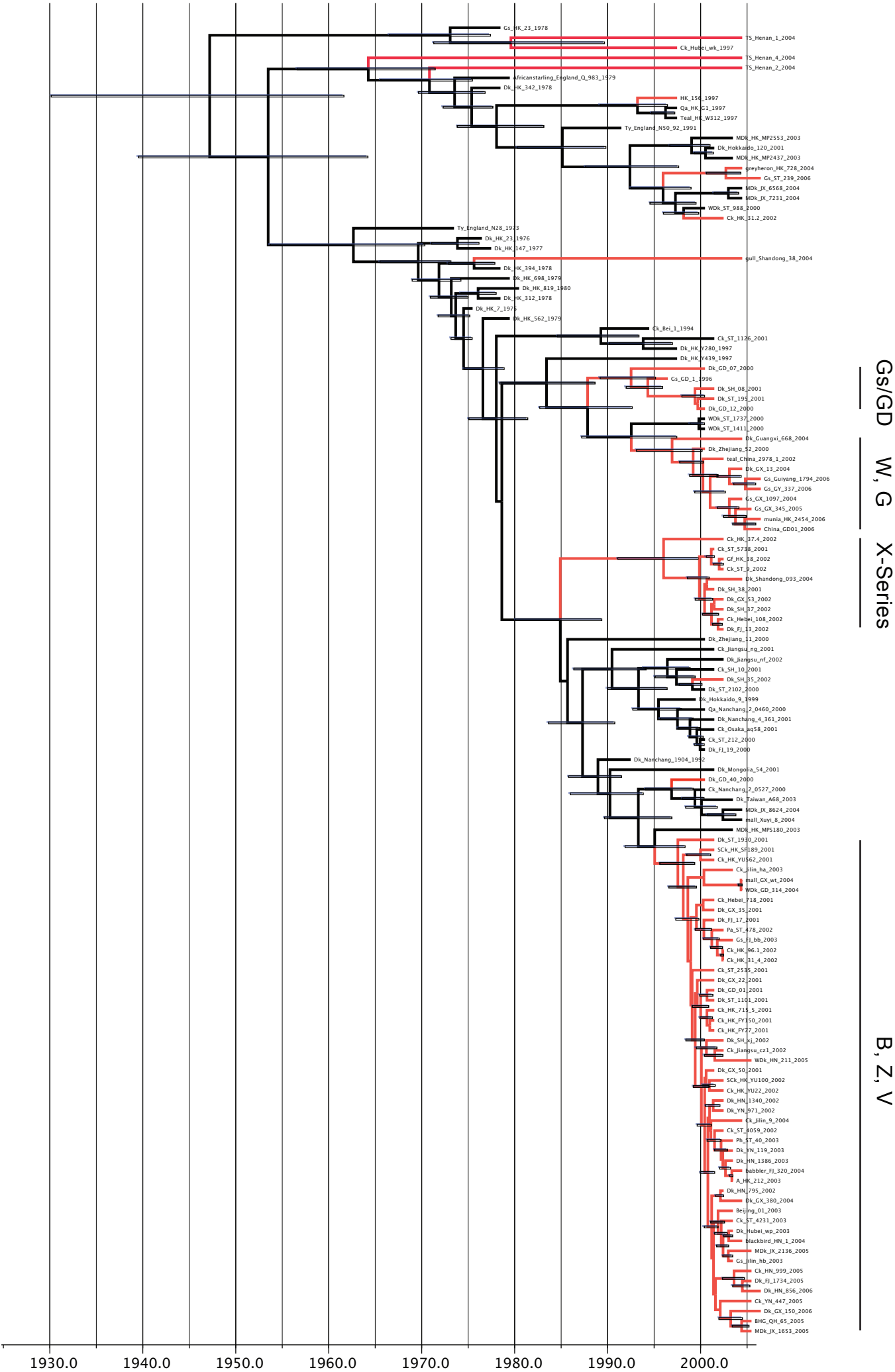

Figure S2 B (PB1)

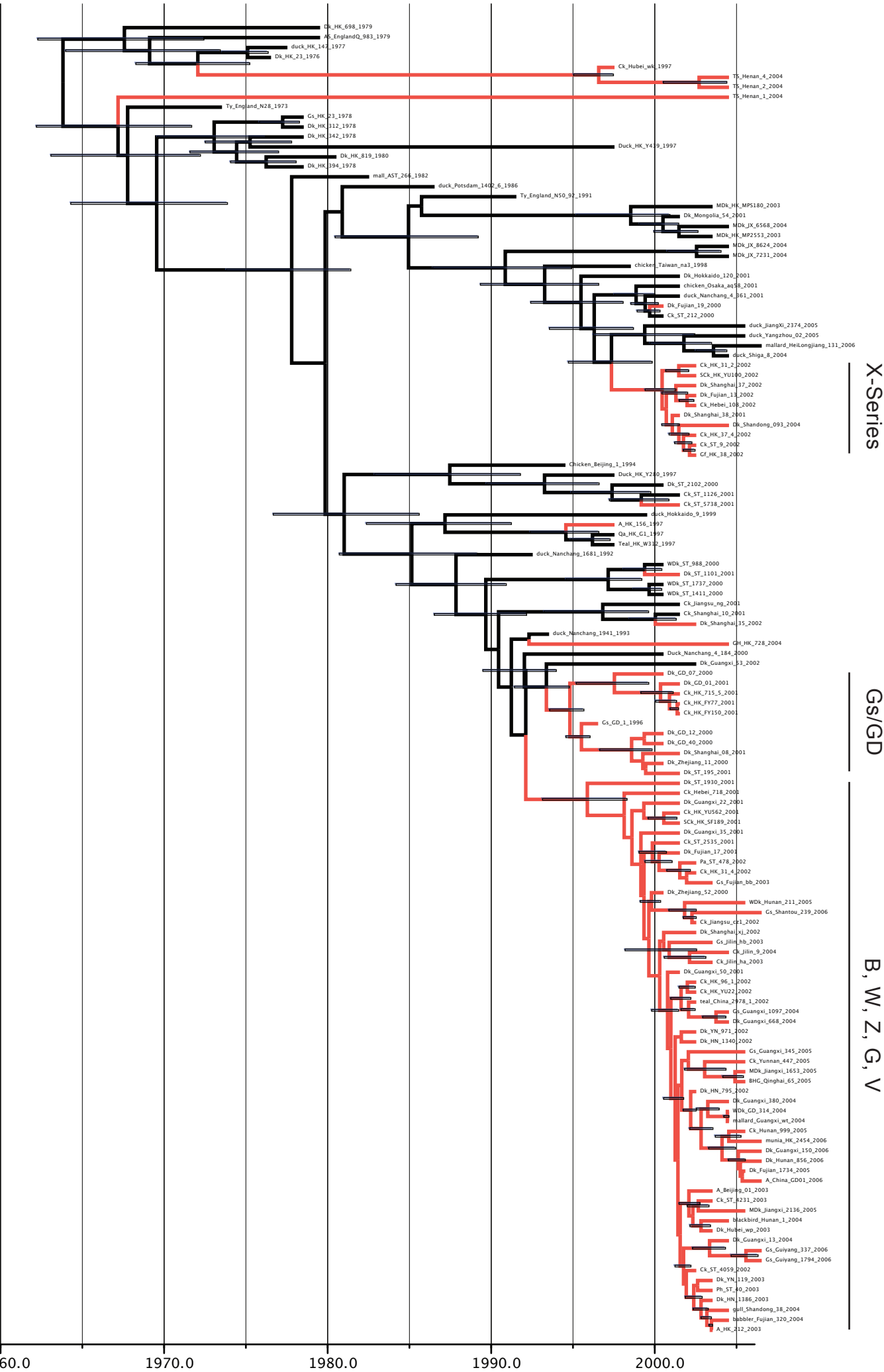

Figure S2 C (PA)

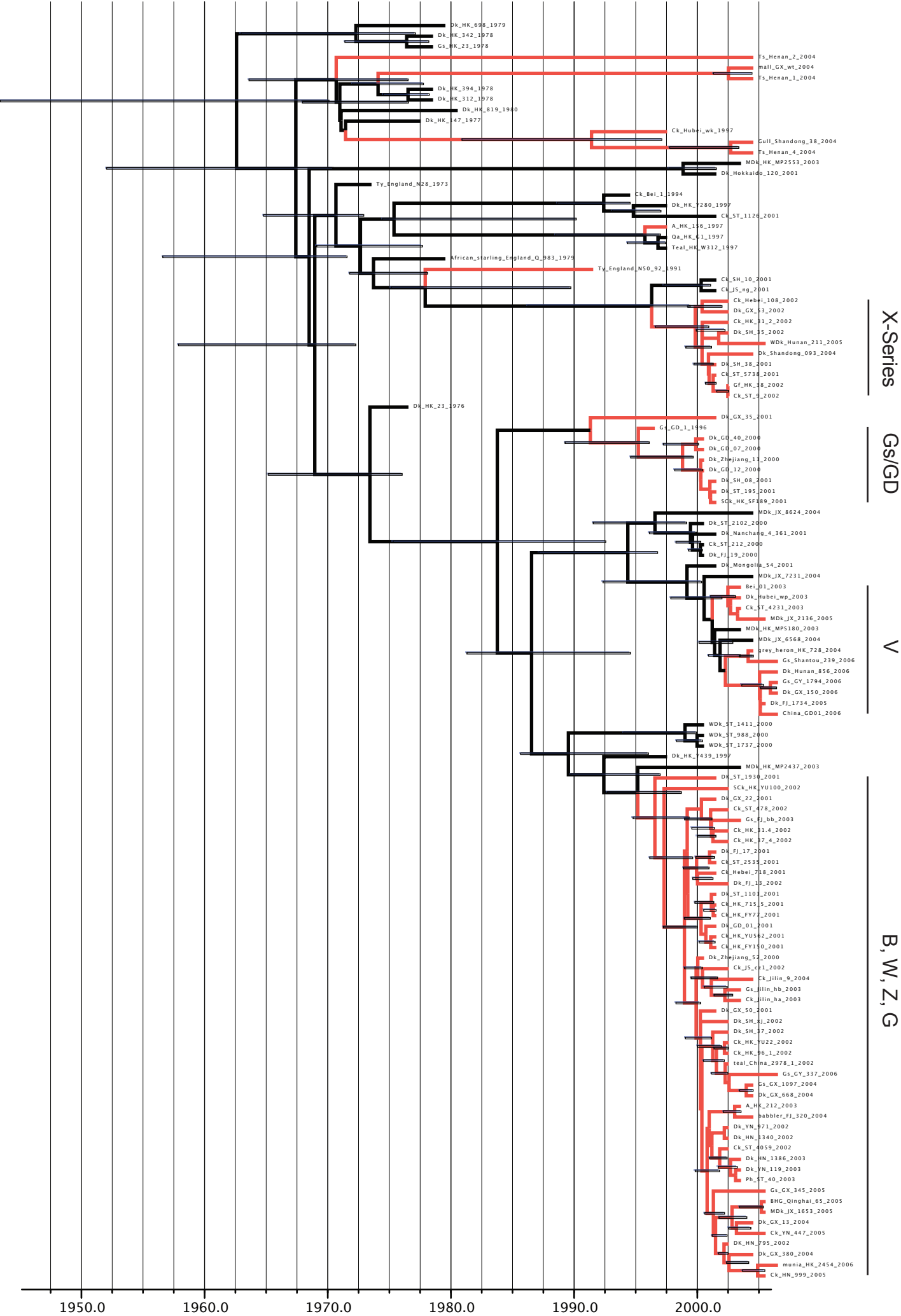

Figure S2 D (NP)

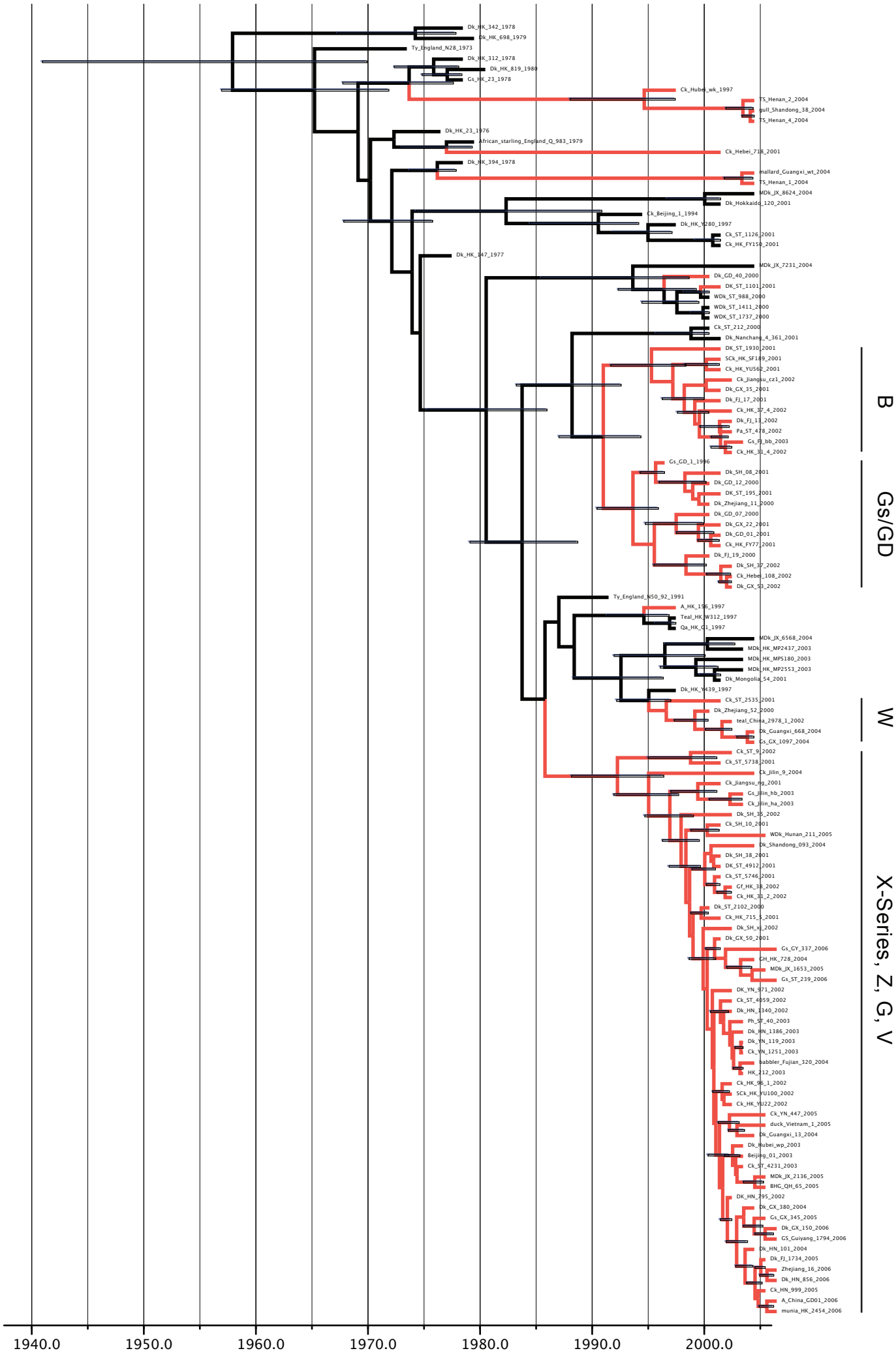

Figure S2 E (M)

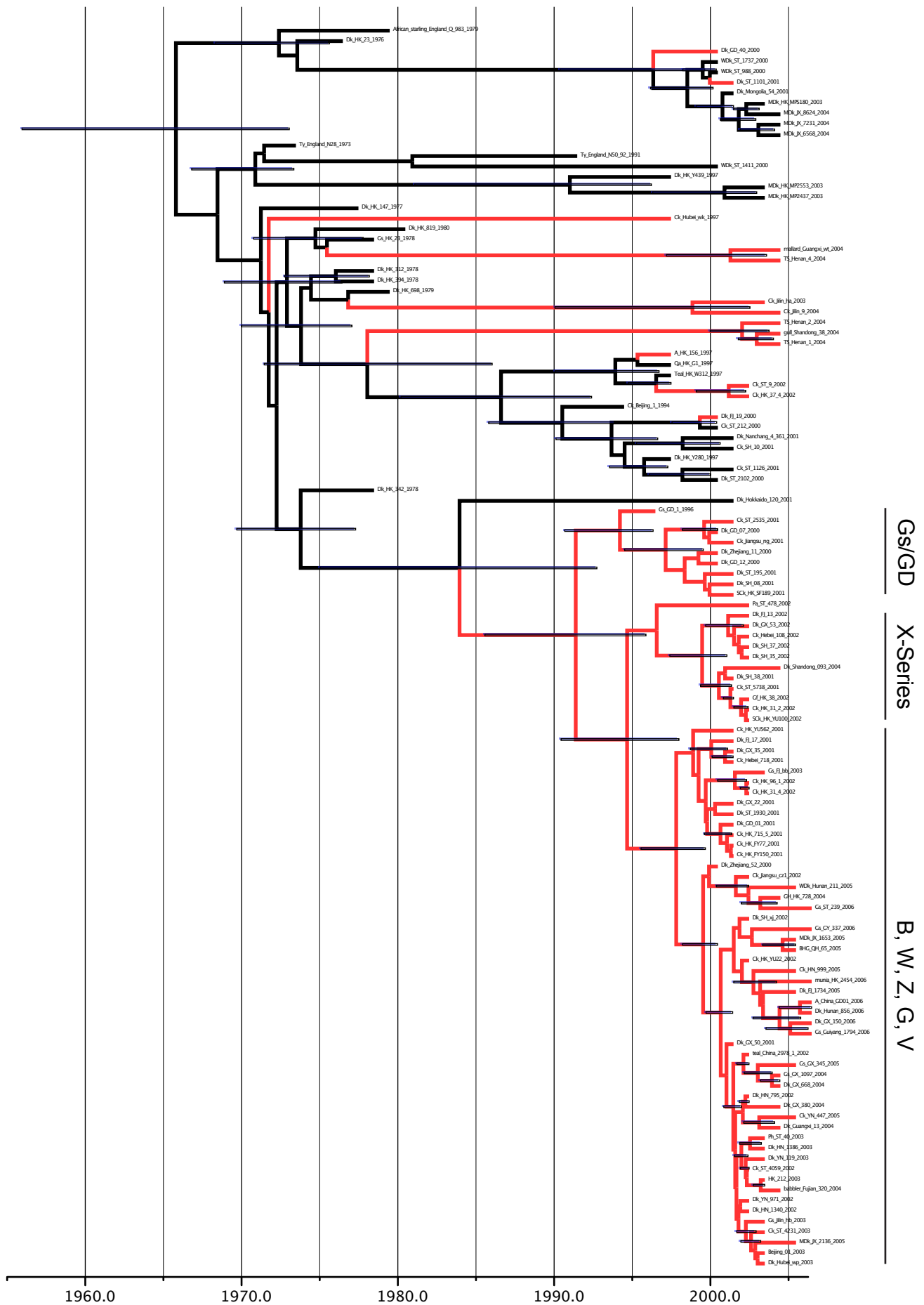

Figure S2 F (NSB)

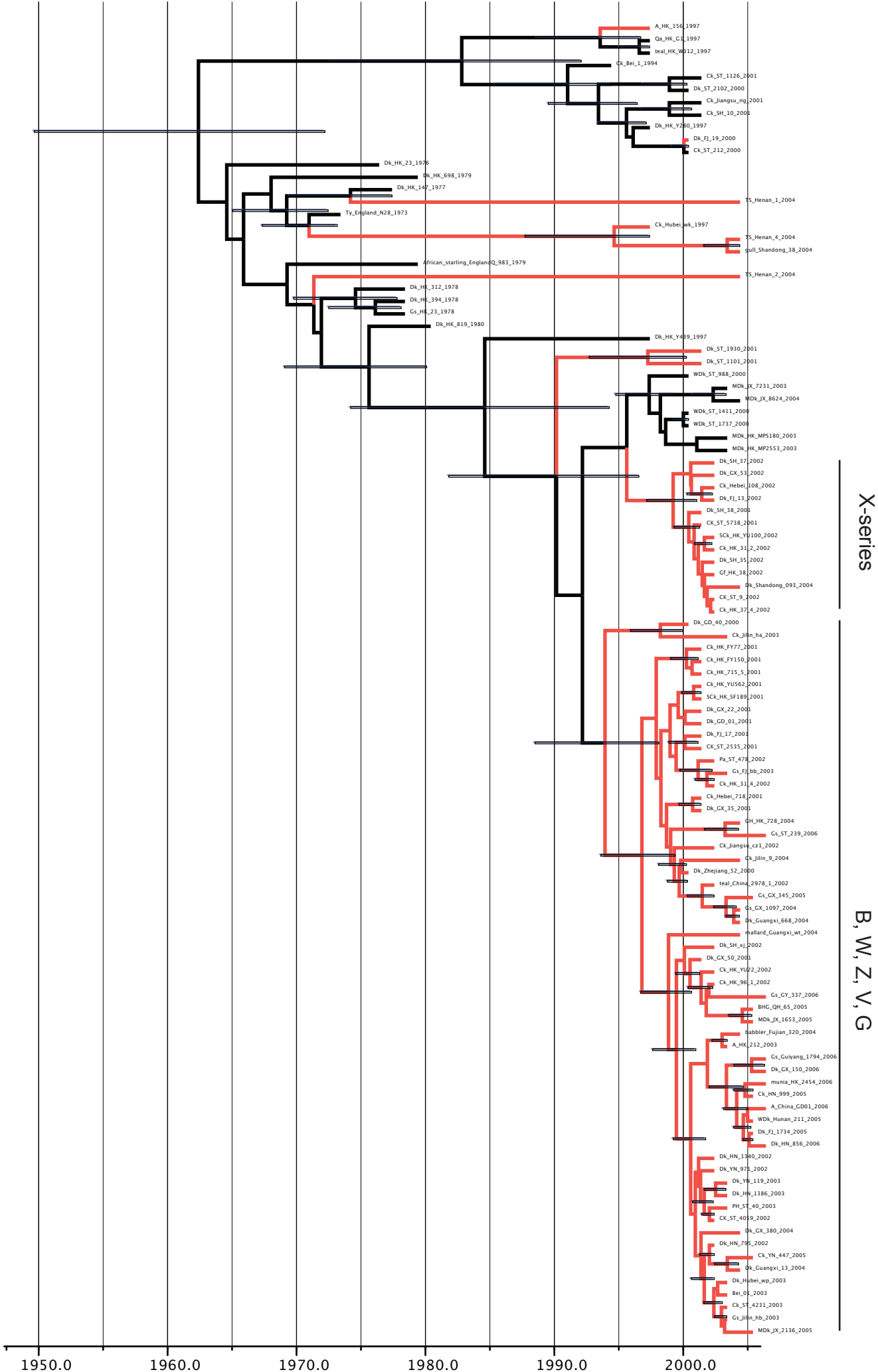

Supplement: Figure S2 — The PB2 (A), PB1 (B), PA (C), NP (D), M (E) and NS (F) gene trees scaled to time (horizontal axis) generated using the SRD06 codon model and uncorrelated relaxed clock model. Nodes correspond to mean TMRCAs and blue horizontal bars at nodes represent the 95% HPDs of TMRCAs. Red branches indicate Gs/GD lineage H5N1 viruses. (1.65 MB PDF) [file ppat.1000161.s002.pdf]

Figure S3

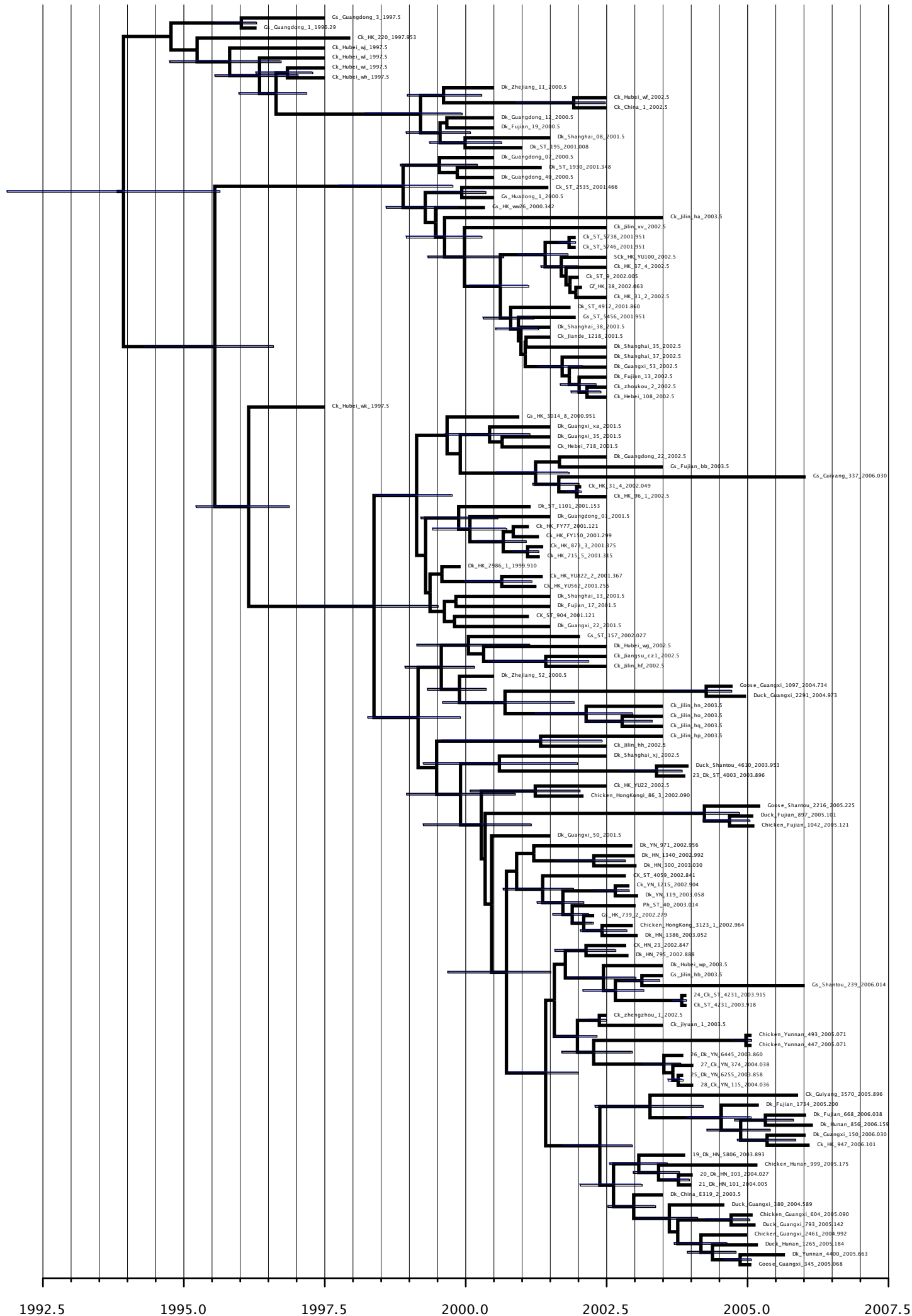

Supplement: Figure S3 — HA gene tree of H5N1 viruses isolated from poultry in China, scaled to time (horizontal axis) generated using the SRD06 codon model and uncorrelated relaxed clock model. Nodes correspond to mean TMRCAs and blue horizontal bars at nodes represent the 95% HPDs of TMRCAs. (0.43 MB PDF) [file ppat.1000161.s003.pdf]
