# Supplementary material for: Evolutionary Dynamics and Emergence of Panzootic H5N1 Influenza Viruses
Source: PLoS Pathog. 2008 Sep 26;4(9):e1000161. doi: 10.1371/journal.ppat.1000161 (PMC2533123; doi:10.1371/journal.ppat.1000161)
Supplement: Table S1 — (0.07 MB PDF) [file ppat.1000161.s004.pdf]

Table S1. To test if the TMRCA of any gene segment (row) of a given genotype is significantly different from the TMRCA of its remaining gene segments (column) a Bayes factor test was conducted*. Bayes factor values that are <0.11 and >9.0 are highlighted in bold. In these cases, the TMRCAs in the comparison are significantly different.

Genotype		PB1	PA	HA	NP	NA	M	NS
GsGD	PB2	1.41	0.71	1.90	0.761	2.64	1.06	6.31
	PB1		0.49	1.42	0.54	2.09	0.83	5.45
	PA			2.49	1.32	1.71	1.52	7.94
	HA				0.37	1.16	0.56	3.01
	NP					3.21	1.21	6.87
	NA						0.47	3.37
	M							5.23
Genotype B	PB2	0.13	0.60		**0**		1.25	**0.03**
	PB1		3.77		**0.03**		**9.17**	0.21
	PA				**0.01**		2.37	**0.06**
	NP						**>>100**	5.06
	M							**0.02**
Genotype X	PB2	**>>100**	3.94		2.57		**9.04**	6.35
	PB1		0.26		**0**		0.65	0.47
	PA				1.76		2.12	1.48
	NP						2.76	0.91
	M							0.70
Genotype W	PB2				**>>100**			

*Bayes factor: $\frac{P(A > B|D)}{1 - P(A > B|D)} \times \frac{1 - P(A > B)}{P(A > B)}$

[Kass and Rafftery (1995), Suchard et al. (2001), Rambaut et al. (2008)].

References

Kass RE, Raftery AE (1995) Bayes factors. J Am Stat Assoc 90: 773–795.

Rambaut A, Pybus OG, Nelson MI, Viboud C, Taubenberger JK, et al. (2008) The genomic and epidemiological dynamics of human influenza A virus. Nature 453: 615–619.

Suchard MA, Weiss RE, Sinsheier JS (2001) Bayesian selection of continuous-time Markov chain evolutionary models. Mol Biol Evol 18: 1001–1013.
